# Supplementary material for: Filamin C dimerisation is regulated by HSPB7
Source: Nat Commun. 2025 May 1;16:4090. doi: 10.1038/s41467-025-58889-x (PMC12046049; doi:10.1038/s41467-025-58889-x)
Supplement: Supplementary file 1 — Supplementary Information [file 41467_2025_58889_MOESM1_ESM.pdf]

Supplementary information for:

Filamin C dimerisation is regulated by HSPB7

Supplementary Methods

Supplementary Figures 1-10

Supplementary Tables 1-4

Supplementary References

Supplementary Methods

Fitting titration data to extract dissociation constants and Gibbs free energies

The titration data for the homo-dimerisation of the various $FLNC_{d24}$ constructs was collected by varying the total concentration of $FLNC_{d24}$ and measuring the ratio of the concentration of dimers to the total concentration. For the hetero-dimerisation of $FLNC_{d24}$ with the different HSPB7 constructs, the total concentration of HSPB7 was kept constant and titrated with varying concentrations of FLNC. The abundances of the different species were extracted from the according peak heights in the mass spectra, and the ratio of the concentration of hetero-dimers to the total concentration of HSPB7 was calculated. The equilibrium constants for the dimerisation reactions were determined by fitting the experimental data to the simplest model for association as follows.

We defined two coupled equilibria for homo- and hetero-dimerisation with associated dissociation constants:

$$FLNC_2 \rightleftharpoons 2\,FLNC, \text{ where } K_{D,homo} = \frac{[FLNC]^2}{[FLNC_2]} \text{ (for homo-dimerisation)}$$

and

$$FLNC{:}HSPB7 \rightleftharpoons FLNC + HSPB7, \text{ where } K_{D,hetero} = \frac{[FLNC][HPSB7]}{[FLNC{:}HSPB7]} \text{ (for hetero-dimerisation).}$$

From the law of mass action, we know that:

$$[FLNC_{total}] = [FLNC] + 2[FLNC_2] \text{ (for homo-dimerisation)}$$

and

$$[FLNC_{total}] = [FLNC] + 2[FLNC_2] + [FLNC{:}HSPB7] \text{ and}$$

$$[HSPB7_{total}] = [FLNC{:}HSPB7] + [HSPB7]$$

(for hetero-dimerisation).

While the expression for the homo-dimerisation data can be solved exactly, that for hetero-dimerisation leads to a cubic equation with non-trivial solutions. We thus solved both numerically using the Scipy *curve_fit* function which uses a non-linear least square fitting protocol. First, we found $K_{D,homo}$ using the *fsolve* function from the *scipy.optimize* module that was used to find the equilibrium concentrations of FLNC monomers and homo-dimers that satisfy these equations for a given value of $K_{D,homo}$ and $[FLNC_{total}]$. The mole fraction of homo-dimers was then calculated, and the optimal value of $K_{D,homo}$ found by fitting the calculated mentioned ratio to the experimental data. This was then used as input for finding $K_{D,hetero}$. Upon knowing $[FLNC_{total}]$, $[HSPB7_{total}]$, $K_{D,homo}$, and $K_{D,hetero}$ the *fsolve* function was used to find the concentrations of $FLNC, FLNC_2, HSPB7$ and $FLNC{:}HSPB7$ that satisfy the law of mass action equations

given above. The optimal value of $K_{D,hetero}$ was found by fitting the calculated fraction of HSPB7 in the hetero-dimer, $[FLNC{:}HSPB7]/[HSPB7_{total}]$ to the experimental data.

The fitted $K_D s$ were used to calculate standard Gibbs free energies using $\Delta G^{\ominus} = -RTlnK_D$, where R is the gas constant, 8.314 $Jmol^{-1}K^{-1}$ and T the temperature, 298.15 K. Errors in as $\Delta G^{\ominus}$ were calculated using error propagation from the errors in fitting the titrations performed to obtain the respective K_D values.

Co-localisation analysis

The plugin JACoP[1], available from https://imagej.net/ij/plugins/track/jacop2.html, accessed 24/04/24) was used on ImageJ 1.53e. Pearson's Coefficient[2] was extracted with the plugin for four images of cryo-sections for each condition. To compare each biomechanical stress condition to its respective control, normally distributed data (Shapiro-Wilk test) were tested by Student’s t-test in GraphPad Prism 10.1.0.

Supplementary Figures

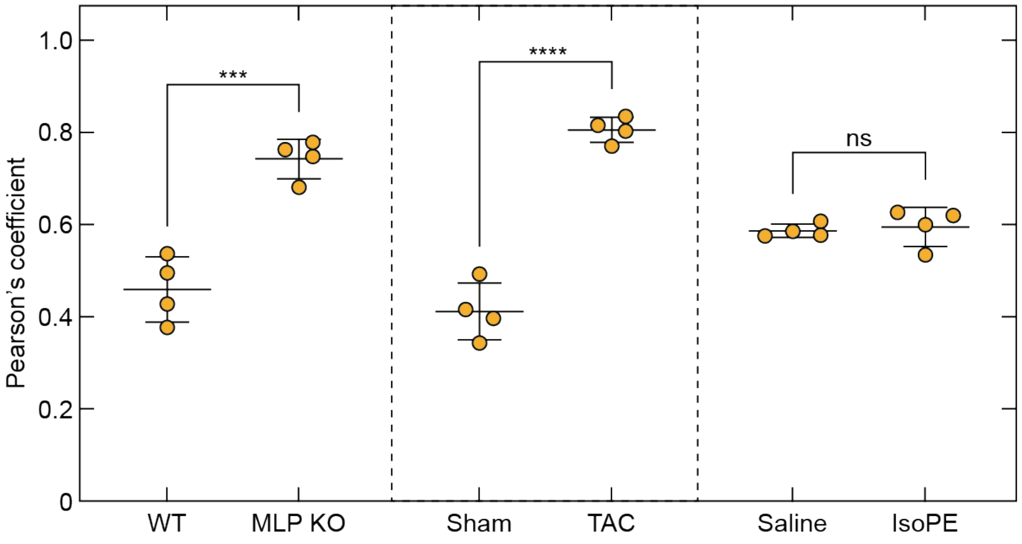

Supplementary Fig. 1 | Co-localisation of HSPB7 and FLNC is increased in two models of biomechanical stress. Four images of from frozen sections of ventricular tissues from TAC, IsoPE and MLP KO mouse models and respective controls (WT, Sham and Saline), stained for HSPB7 and FLNC, were analysed for colocalisation with JACoP plugin for ImageJ. The Pearson's Coefficient for signals of both proteins is shown (mean ± standard deviation). The biomechanical stress condition was compared to its respective control by Student's t test, *** $p < 0.001$, **** $p < 0.0001$. ns – not significant ($p > 0.05$). Please note, in the Saline – IsoPE pair of biomechanical stress, the Saline controls already have elevated Pearson's Coefficients (~0.6 versus ~0.4 for the other two control settings). This indicates stress in this control condition through the implantation of a saline-releasing osmotic minipump, which is also reflected in increased signal of HSPB7 at the intercalated discs in this setting compared to the other two control settings (WT and Sham, **Fig. 1C**).

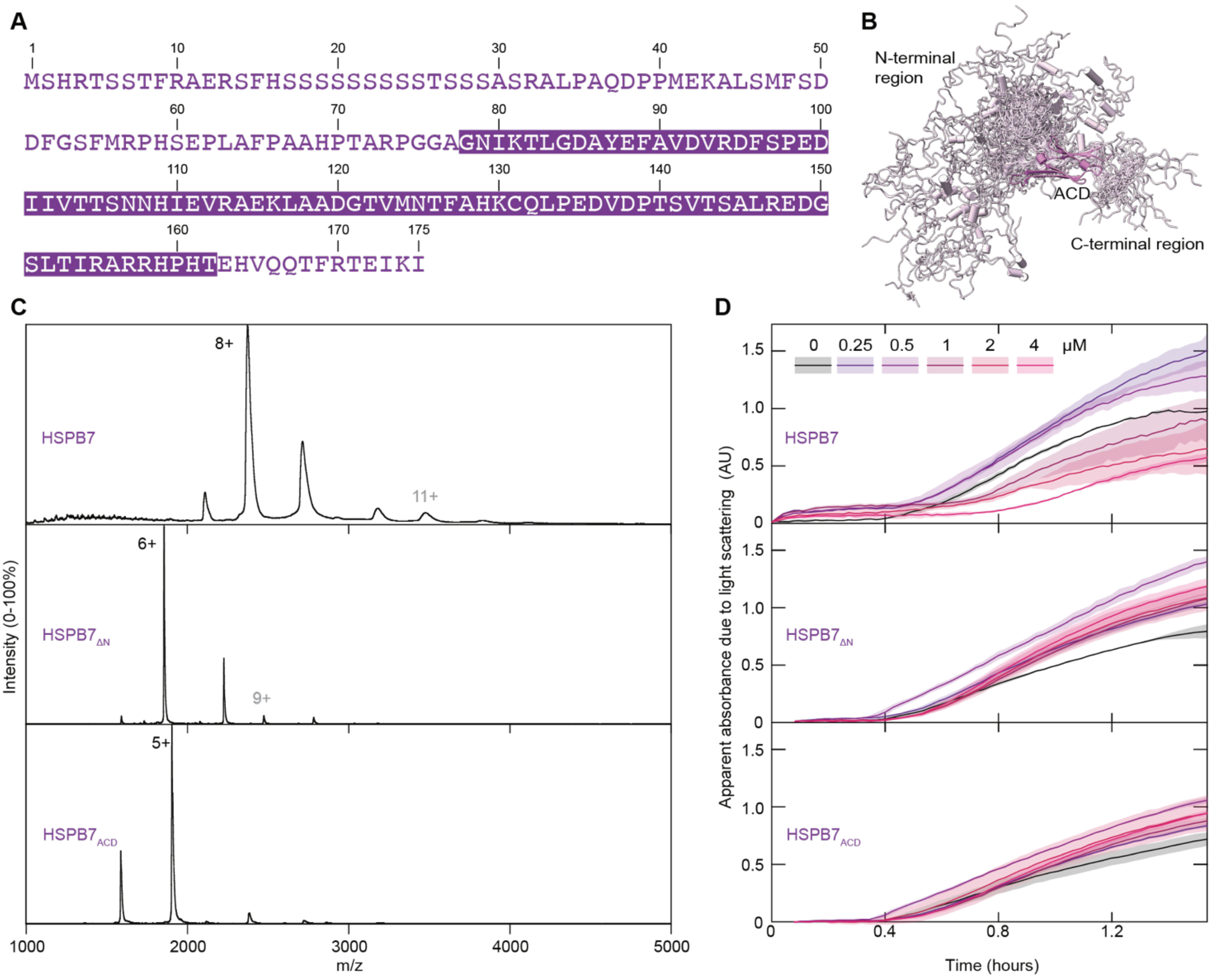

Supplementary Fig. 2 | HSPB7 is predominately monomeric with weak chaperone activity. A The sequence of HSPB7 (Uniprot ref: Q9UBY9-2, Isoform 2, A67 to AAHPTA, 175 residues), with the sequence for the ACD construct used in this work highlighted. **B** An ensemble of computational predictions using AlphaFold for the full-length HSPB7 structure, aligned to the structure of HSPB7$_{ACD}$, show the N- and C-terminal regions are likely disordered and heterogeneous. **C** Native mass spectra of the HSPB7 constructs at 10 µM show monomers (black charge state label) and only extremely low abundances of dimer (grey charge state) in all cases. **D** Chaperone assays in which the ability of HSPB7, HSPB7$_{\Delta N}$ and HSPB7$_{ACD}$ to attenuate heat-induced aggregation of citrate synthase (CS) was tested. The black curve reports the apparent absorbance due to light scattering due to the aggregation of 1 µM CS (without the addition of any of the HSPB7 constructs). Addition of HSPB7$_{\Delta N}$ and HSPB7$_{ACD}$, at concentrations up to 4 µM (purple to pink curves), does not decrease the aggregation of CS. Instead the apparent absorbance is somewhat increased, consistent with a weak interaction between the proteins leading to co-aggregation. For HSPB7, at the highest concentrations, the aggregation of CS was somewhat attenuated. These results show that the chaperone activity of HSPB7 is very limited, and relies heavily on the N-terminal region.

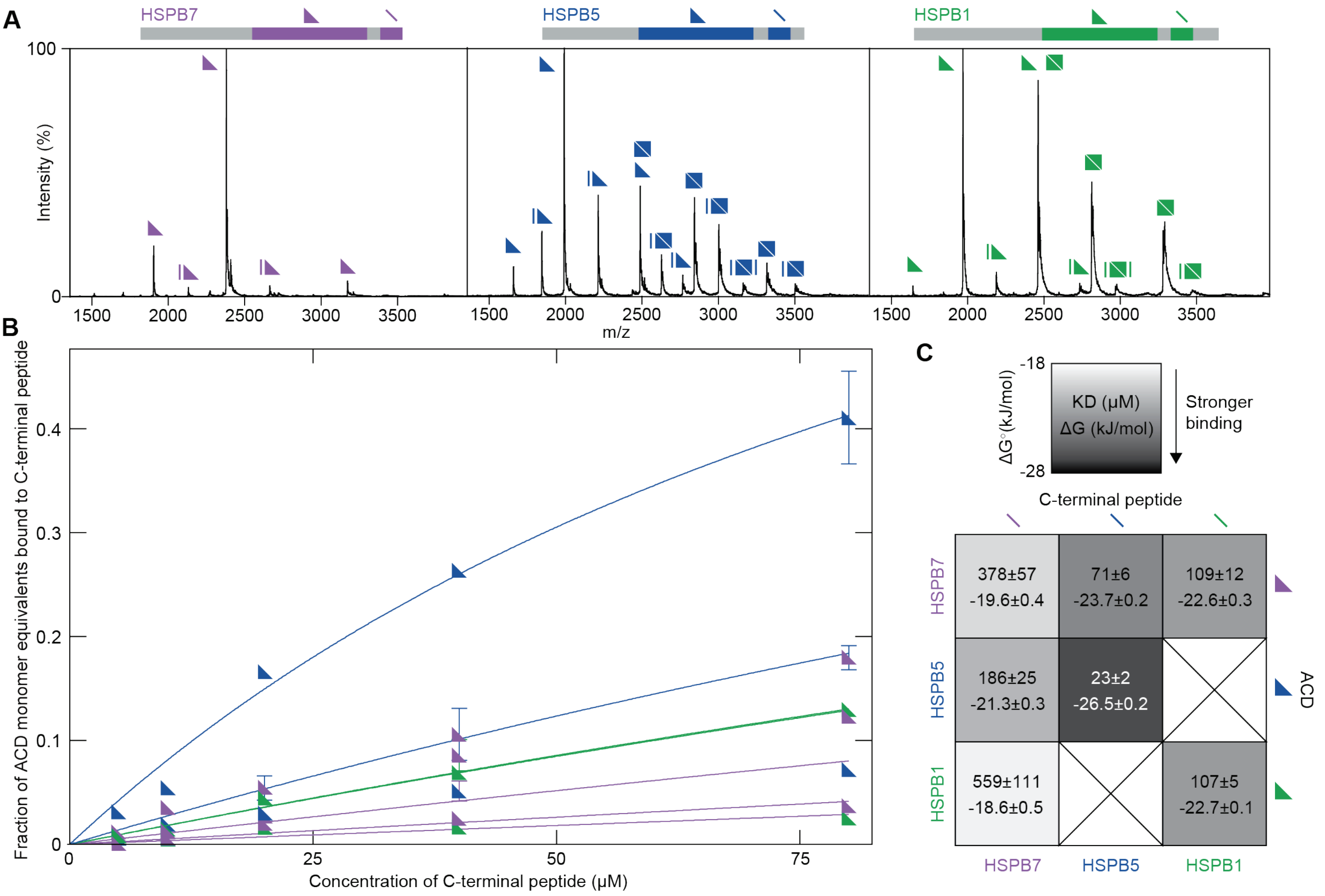

Supplementary Fig. 3 | The HSPB7 C-terminal region does not bind to sHSP ACDs. A Native mass spectra obtained when 5 µM of sHSP (HSPB7, purple; HSPB5, blue; HSPB1, green) ACD (triangle) were incubated with 40 µM of a peptide with their C-terminal sequence containing the IXI motif (line). The binding between $HSPB7_{ACD}$ and its own C-terminal peptide is very weak, compared with the same interaction in HSPB1 and HSPB5. **B** The titration experiments of the sHSP ACD-peptide interactions, the protein concentrations were maintained at 5 µM. The colours of the triangular symbols and line represent, respectively, the ACD peptide used in the titration (HSPB7, purple; HSPB5, blue; HSPB1, green). Datapoints represent the mean of technical replicates (n=3), and the error bars ±1 SD. **C** The extracted thermodynamic parameters of sHSP ACD-peptide interactions. The matrix is shaded according to the strength of binding, and shows that the root cause of HSPB7 not binding its C-terminal peptide to a significant extent lies in the C-terminal, not in the ACD, sequence.

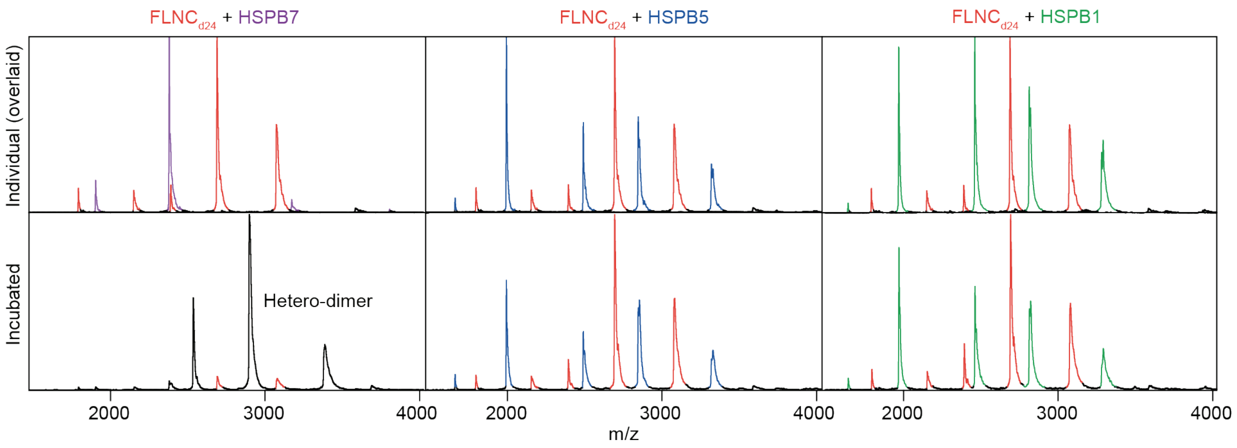

Supplementary Fig. 4 | FLNC$_{d24}$ binds HSPB7$_{ACD}$ specifically. Native MS data of FLNC$_{d24}$ (red) incubated with HSPB7$_{ACD}$ (purple), HSPB5$_{ACD}$ (blue) or HSPB1$_{ACD}$ (green). The upper row shows the spectra of the individual proteins overlaid, the bottom row shows the interactions between proteins mixed at the monomer ratio of 1:1. Only HSPB7 forms observable amounts of hetero-dimer (black) with FLNC$_{d24}$. Experiments were performed under reducing conditions, given the ability of HSPB1 to form a covalently linked ACD homo-dimer.

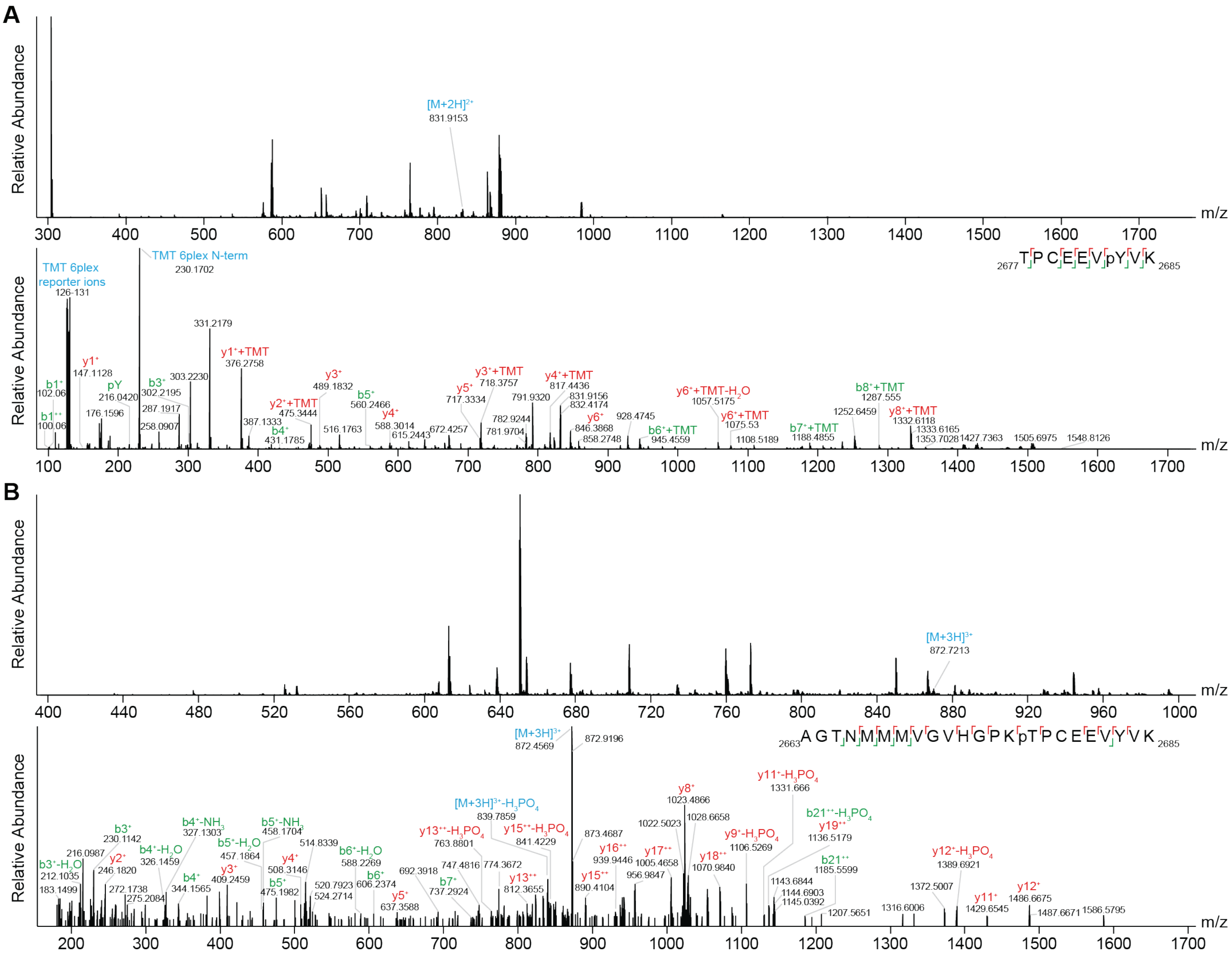

Supplementary Fig. 5 | Annotated mass spectra of tryptic phosphopeptides of FLNC validate phosphorylation at T2677 and Y2683. A MS1 mass spectrum (upper panel) with precursor peptide containing pY2683 selected for tandem-MS analysis indicated. The resulting MS2 mass spectrum (lower panel) displays many fragment ions, with those assignable to *b*- (green) and *y*-ions (red) annotated. The ion series provide phosphosite-specific information for the unambiguous localisation of pY2683. Data was extracted from the PRIDE database, having been deposited by Hoffmann et al[3] (PDX001543). TMT 6plex labeling was used in the study, hence TMT reporter ions and uncleaved TMT labels are also labelled. **B** MS1 mass spectrum (upper panel) with precursor peptide containing pT2677 selected for tandem-MS analysis indicated. The resulting "MS2" mass spectrum (lower panel) displays many fragment ions, with those assignable to *b*- (green) and *y*-ions (red) annotated. Fragment ions with a neutral loss of phosphoric acid (-H_3PO_4), ammonia (-NH_3), or water (-H_2O) are indicated in the MS2 spectra. The ion series provide phosphosite-specific information for the unambiguous localisation of pY2677. Data was extracted from the PRIDE database, having been deposited by Bouhaddou et al[4] (PDX019113).

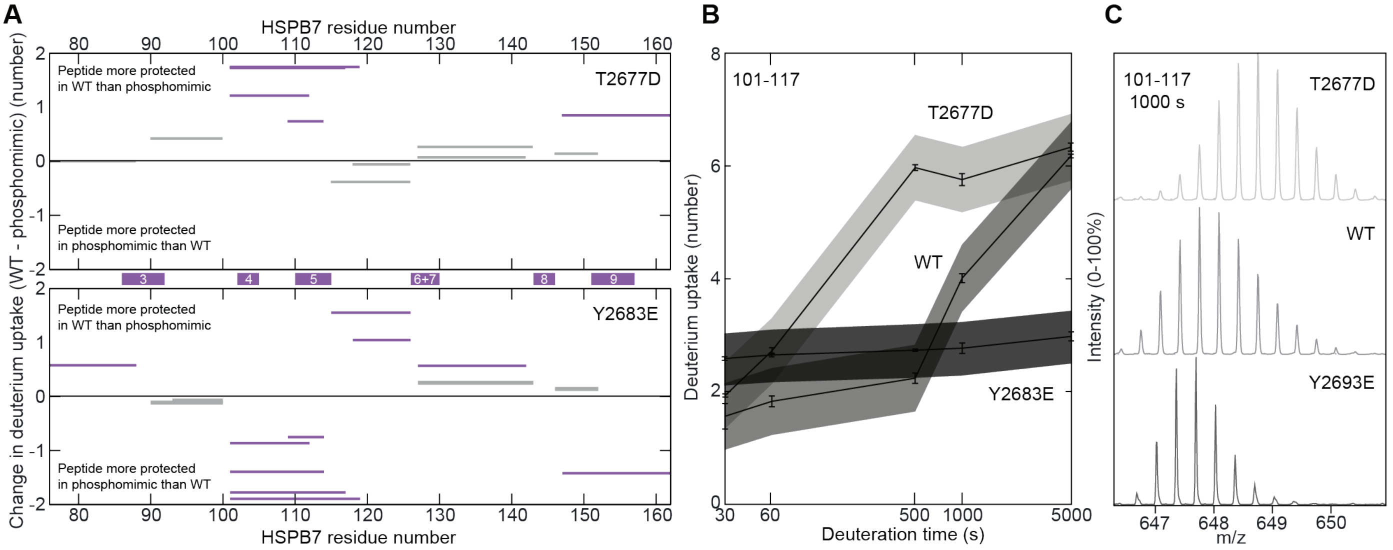

Supplementary Fig. 6 | HDX reveals differences in solvent accessibility upon mimicking phosphorylation at the hetero-dimer interface. A The Woods plots showing the differential deuterium uptake (after 1000 s of labelling) of $HSPB7_{ACD}$ hetero-dimerised with WT $FLNC_{d24}$, or one of the two phosphomimics T2677D (upper panel) and Y2683E. **B** A plot of deuterium uptake against deuteration time of the most protected peptide on $HSPB7_{ACD}$ (residues 101-117) in the different hetero-dimers. T2677D is the least protected, and Y2683E the most protected. This agrees with our native MS data that showed a weakening of the hetero-dimer interface in the former, and a strengthening in the latter, compared to the WT (**Fig. 4**). Error bars refer to the standard deviation of three repeats at each time-point, and the shaded bands a 99% confidence band. **C** Raw mass spectra of the $HSPB7_{ACD}$ peptide (residues 101-117), in the 3+ charge state at 1000 s, for the each of the three complexes shows how the differences in deuteration are readily visible by eye.

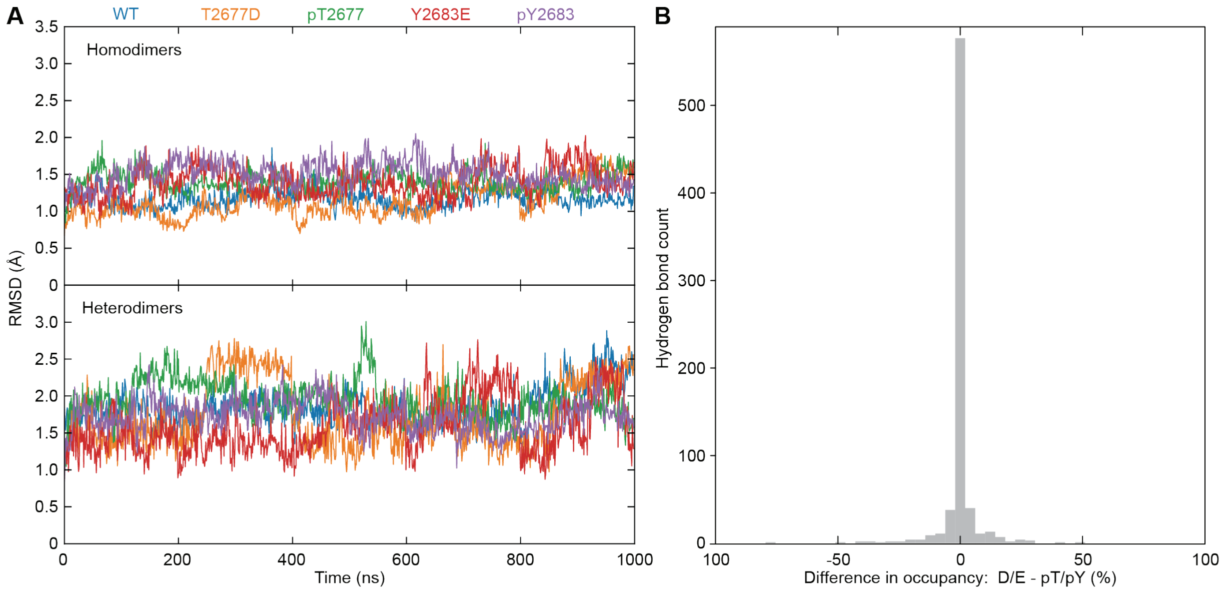

Supplementary Fig. 7 | MD simulations are stable, and show little difference between phosphorylated and phosphomimicking forms. A Each of the simulated systems (homo- and hetero-dimers, for each of the WT, pT2677, T2677D, pY2683, Y2683E) remained stable over the 1-µs production run. In every case, the RMSD of the production run (compared to the first frame) does not show a systematic trend of increase over time. For homo-dimers, the RMSDs are always <2 Å (with fluctuations <0.5 Å), whereas for hetero-dimers the RMSD is always <2.9 Å (with fluctuations <1.5 Å). **B** To assess whether the mutations T2677D and Y2683E are good mimics of the actual phosphorylation we have performed four new MD simulations. We obtained 1-µs simulations on T2677D and Y2683E FLNC homo- and hetero-dimers (mutations modelled into the WT structure using the Mutator plug-in for VMD), to compare with those for pT2677 and pY2683. We examined the 368 hydrogen bonds that were observed in the simulation of at least one FLNC form, and assessed the differences in occupancy between the phosphomimic and phosphorylated form for each (T2667D − pT2677, and Y2698 − pY2683). Of the 736 hydrogen bond occupancy difference instances assessed from these simulations, 598 (81.2%) showed differences in occupancy lower than ±2%, while 57 (7.7%) exceeded ±10% difference, and 22 (3.0%) exceeded ±20%. This indicates that phosphomimicking and phosphorylation forms at sites 2677 and Y2683 have very similar impact on FLNC *in silico*, with no significant overall changes in structure between them. Examining next the 28 hydrogen bond occupancy difference instances that involve sites 2677 and 2683 directly (as either the bond donor or acceptor), we find that 21 (75%) showed differences in occupancy lower than ±2%, while 5 (18%) exceeded ±10% difference, and 3 (11%) exceeded ±20%. This demonstrates that even in the direct contacts made at sites 2677 (whether by pT or D), or at 2683 (whether by pY or E), the occupancies are very similar. We note that for 4 of the 5 hydrogen bonds that showed a difference in occupancy exceeding ±10%, that they were more occupied in the phosphorylated than phosphomimicking forms. This is consistent with the Cα-O distances: each phosphorylated residue is longer than its phosphomicking equivalent (pT=3.7 Å > D=3.3 Å and pY=7.7 Å > E=4.6 Å) and therefore has longer "reach"; and the phosphate groups (in pY and pT) carried two negative charges while the carboxylate groups (in D and E) only carried one. Both effects can act to make a particular bond stronger in the case of phosphorylation versus phosphomimicry. All hydrogen bond occupancy data is available in the **Supplementary Data 1**.

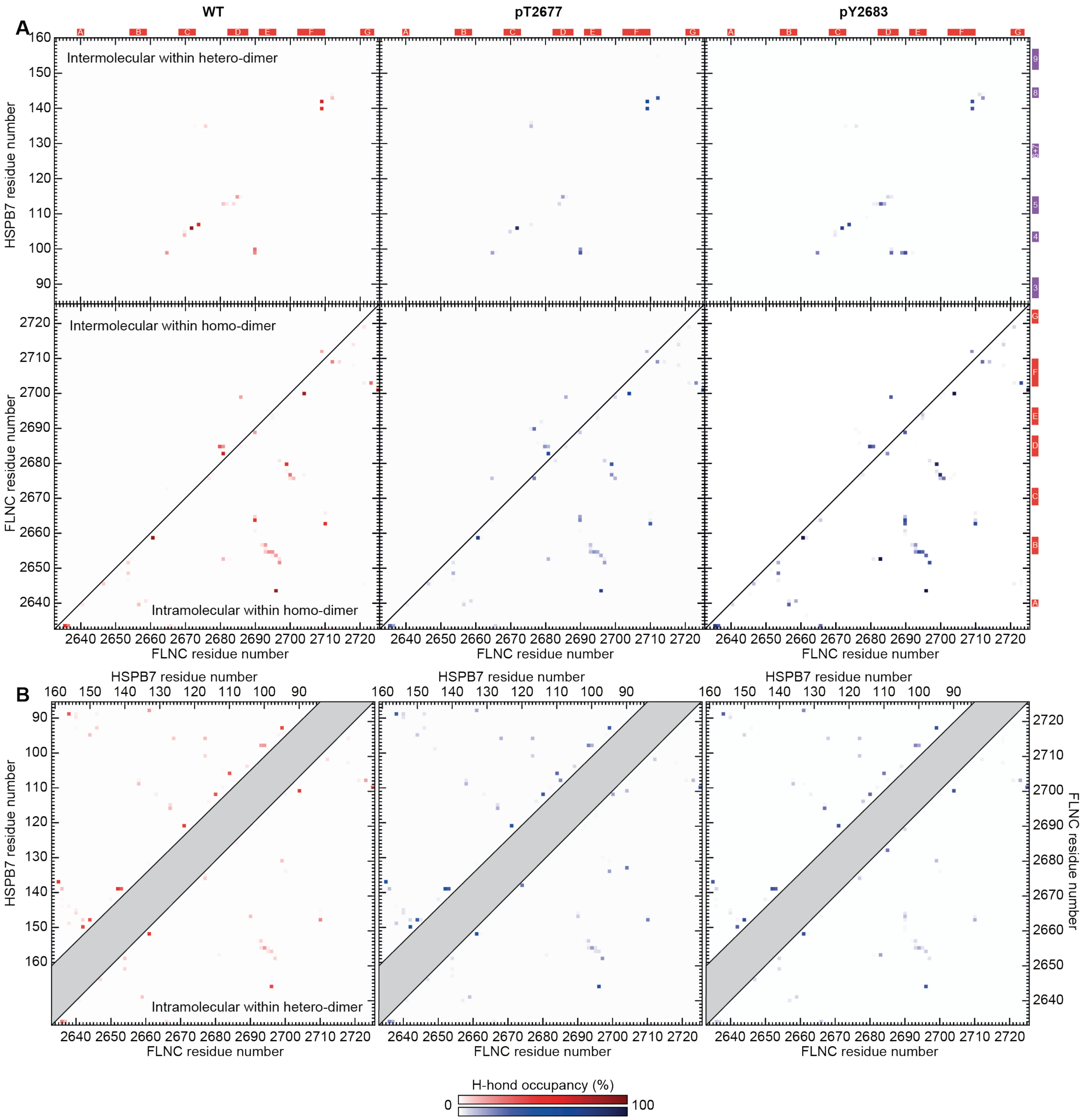

Supplementary Fig. 8 | Hydrogen-bond maps for WT and phosphorylated FLNC$_{d24}$ homo- and hetero-dimers. The data underlying the difference maps shown in **Fig. 5** and **Supplementary Fig. 9**. **A** Bond occupancy for inter-molecular hydrogen bonds within the hetero-dimer (upper row), and intra-molecular within the homo-dimer (lower row), for WT, pT2677 and pY2683 (from left to right). The darker the pixel, the larger the percentage of frames within the MD simulation in which a hydrogen bond is formed between those two residues. **B** Bond occupancy for intra-molecular hydrogen bonds within the hetero-dimer. Bonds within HSPB7 are shown in the upper left triangles, and bonds within FLNC are shown in the lower right triangles. Data for WT, pT2677 and pY2683 (from left to right) are displayed using the same colour scale as for **A** (shown at the bottom).

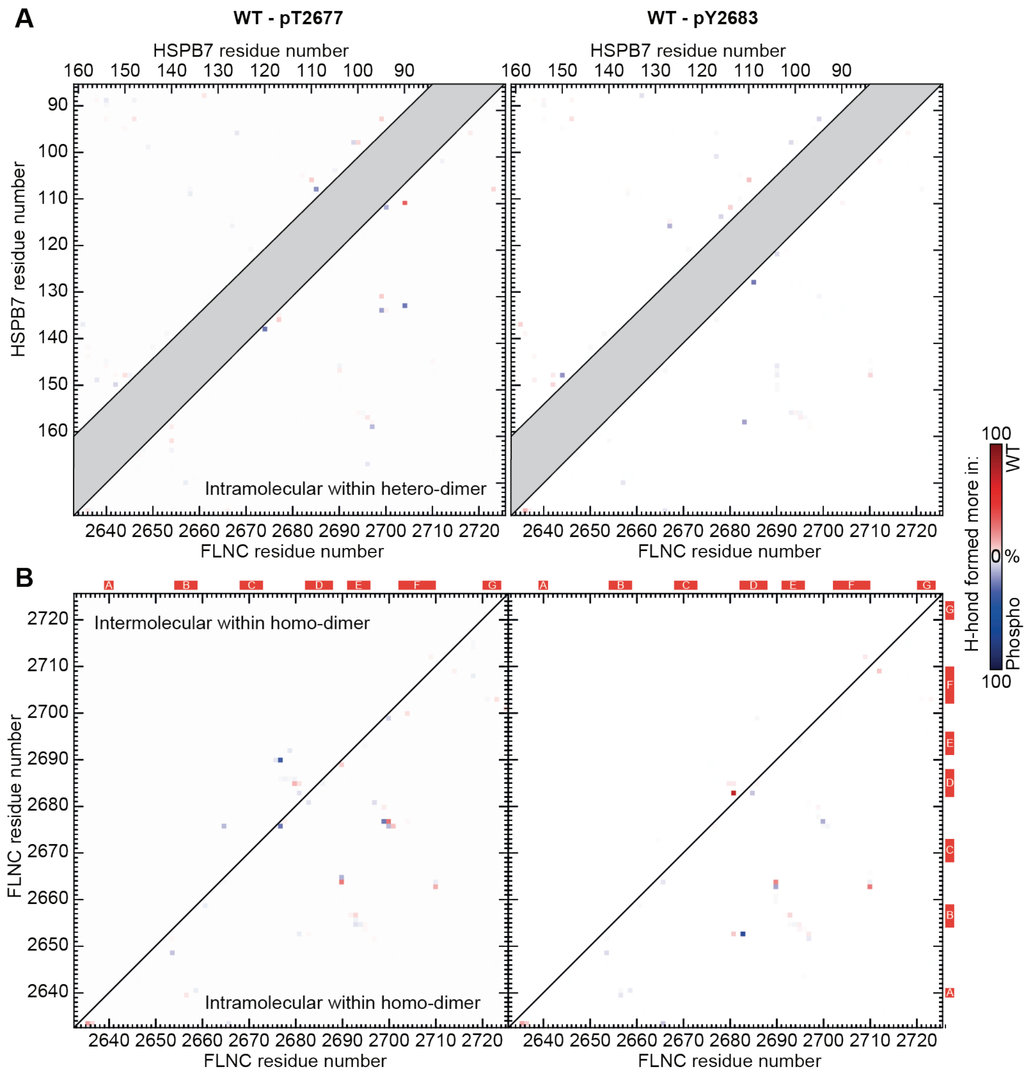

Supplementary Fig. 9 | Hydrogen-bond difference maps for WT and phosphorylated FLNC$_{d24}$ homo- and hetero-dimers. A Difference in bond occupancy for intra-molecular hydrogen bonds within the hetero-dimer between WT and pT2677 (left) or pY2683 (right). Bonds within HSPB7 are shown in the upper left triangles, and bonds within FLNC are shown in the lower right triangles. **B** Difference in bond occupancy for inter-molecular (upper left triangles) and intra-molecular (lower right triangles) hydrogen bonds within the homo-dimer between WT and pT2677 (left) or pY2683 (right). Colour scale as for **A** (shown to the right).

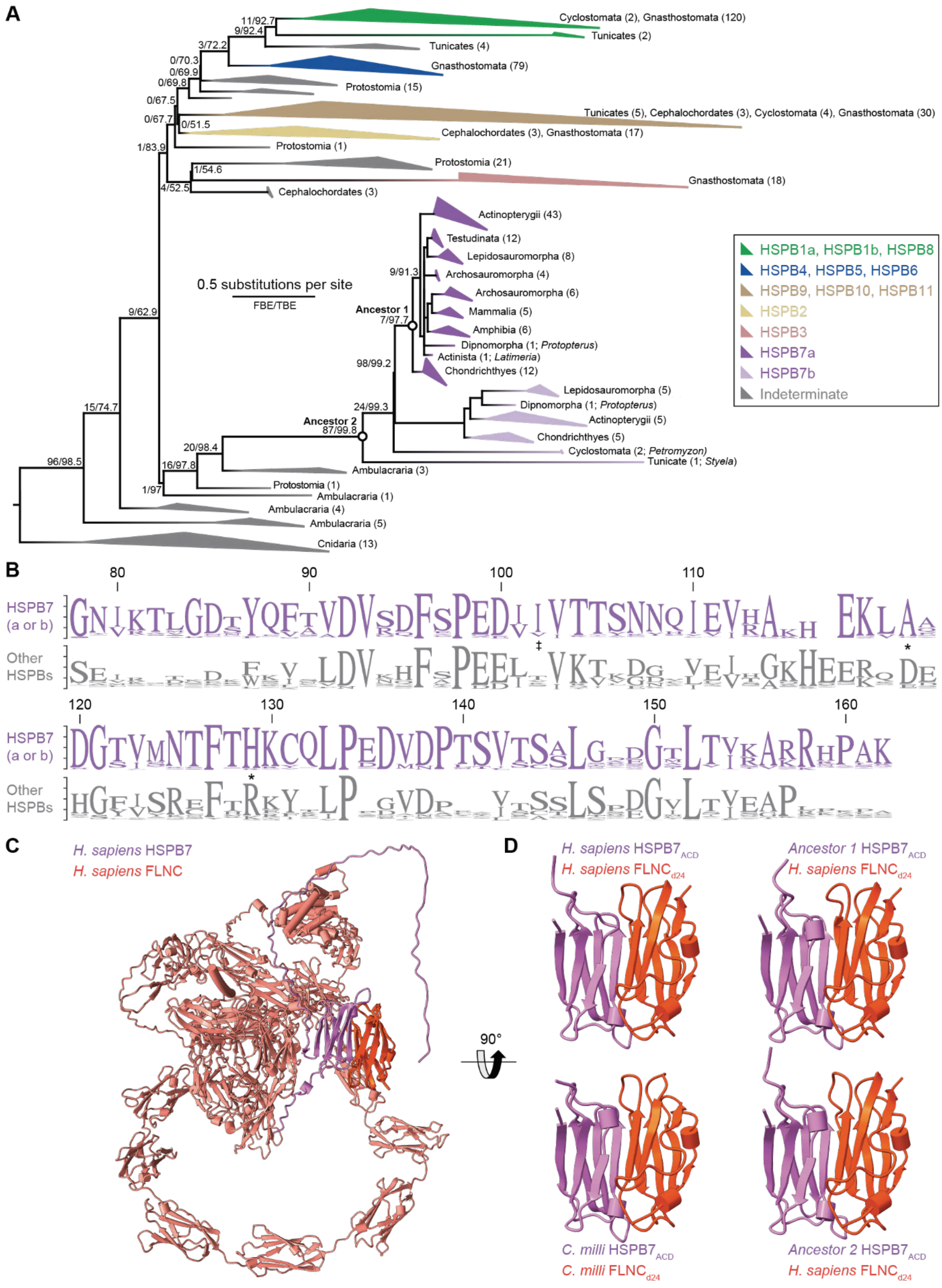

Supplementary Fig. 10 | The interaction between FLNC and HSPB7 is ancient. A Maximum likelihood phylogeny of HSPBs based on searching for all of HSPB1-11. HSPB7 can be identified in species as distantly related to humans as tunicate *Styela*, and is present in all vertebrate clades in between. We find two distinct forms of HSPB7, which we term HSPB7a and HSPB7b, with humans having only the first type. Ancestral proteins that we reconstructed are indicated at their corresponding nodes on the tree. **B** Sequence logo of the frequency of different amino acids in the ACD for all the HSPB7s and other HSPBs (grey) in our phylogeny. The two sites we identified (**Fig. 2C**) as likely responsible for monomerisation are marked (*), as is the site of I102 (in human HSPB7) that is involved in the hydrophobic interface of the hetero-dimer (‡). **C** AlphaFold

model of a single chain of full-length human FLNC (orange; d24 in a darker hue) in complex with a single chain of HSPB7 (purple). The interface we observed in our structure of $FLNC_{d24}$:$HSPB7_{ACD}$ (**Fig. 3B**) is preserved in this model, demonstrating that the missing domains of FLNC and terminal regions of HSPB7 do not preclude hetero-dimerisation. **D** AlphaFold models of the $FLNC_{d24}$:HSPB7 hetero-dimer for the human and *C. milli* proteins (left), and the human $FLNC_{d24}$ with ancestral forms of HSPB7 (right) in our competitive binding assay. Only one chain of each protein is shown in these models, and the terminal regions of HSPB7 are hidden, for clarity. All four cases form hetero-dimers that align essentially perfectly with our crystal structure (**Fig. 3B**).

Supplementary Tables

	HSPB7$_{ACD}^{C131S}$	FLNC$_{d24}$:HSPB7$_{ACD}^{C131S}$
**PDB ID**	8RHA	8PA0
**Data collection**		
Space group	$P4_12_12$	$P3_221$
Cell dimensions		
a, *b*, *c* (Å)	57.86, 57.86, 167.34	94.13, 94.13, 51.60
α, β, γ (°)	90, 90, 90	90, 90, 90
Synchrotron beamline	DLS I04	DLS I03
Data collection temperature (K)	100	100
Wavelength (Å)	0.97950	0.97950
Resolution (Å)	55.78-2.18 (2.22-2.18) *	47.04-2.85 (2.90-2.85) *
No. total reflections	398937 (19156)	122811 (6265)
No. unique reflections	15713 (764)	6383 (316)
R_{merge}	0.138 (1.418)	0.288 (2.487)
$I / \sigma I$	14.5 (2.2)	6.1 (1.9)
Completeness (%)	100 (99.1)	100 (100)
Redundancy	25.4 (25.1)	19.2 (19.8)
CC1/2	1 (0.957)	0.995 (0.654)
**Refinement**		
Resolution (Å)	54.68-2.18 (2.22-2.18)	47.06-2.85 (3.59-2.85)
R_{work} / R_{free}	0.2534/0.2796	0.2243/0.2909
No. atoms		
Protein	1,807	1,350
Ligand/ion	0	0
Water	6	4
Average *B*-factors <Å^2>		
Protein	62.78	59.93
Ligand/ion	/	/
Waters	46.06	51.37
R.m.s. deviations from ideal		
Bond lengths (Å)	0.006	0.005
Bond angles (°)	1.399	0.54
Ramachandran favored (%)	97.33	94.19
Ramachandran allowed (%)	2.67	5.81
Ramachandran outliers (%)	0.00	0.00

*Values in parentheses are for highest-resolution shell.

Supplementary Table 1 | Data collection and refinement statistics for HSPB7$_{ACD}^{C131S}$ and FLNCd$_{24}$:HSPB7$_{ACD}^{C131S}$.

	Theoretical (Da)	Experimental (Da)
$FLNC_{d24}$ monomer	10747.4	10758.7±1.4
$FLNC_{d24}$ dimer	21494.8	21519.4±0.7
$FLNC_{d24}^{T2677D}$ monomer	10761.4	10775.5±0.7
$FLNC_{d24}^{T2677D}$ dimer	21522.8	21544.9±1.4
$FLNC_{d24}^{Y2683E}$ monomer	10713.4	10723.5±2.1
$FLNC_{d24}^{Y2683E}$ dimer	21426.7	21447.4±0.7
$HSPB7_{ACD}$	9507.5	9517.2±1.2
$FLNC_{d24}$:$HSPB7_{ACD}$	20254.9	20277.6±3.2
$FLNC_{d24}^{T2677D}$:$HSPB7_{ACD}$	20268.9	20294.4±2.1
$FLNC_{d24}^{Y2683}$:$HSPB7_{ACD}^{E}$	20220.9	20241.9±2.8

Supplementary Table 2 | Theoretical and experimental masses of the complexes. Theoretical masses are calculated directly from the sequence. Experimental masses were obtained from native MS, and are measured from the peak centres. Native MS data were obtained under soft desolvation conditions so as not to artefactually dissociate the complexes, which leads to residual binding of buffer components. Consequently, somewhat higher masses (typically <1%[5]) are measured than predicted by sequence alone. However, these mass shifts are small relative to the mass differences between our assignments and any other alternatives given the few components in each mixture.

	$HSPB7_{ACD}$	$FLNC_{d24}$	Distance (Å)
**Salt bridges**	D100 OD1	H2686 ND1	2.64
	D100 OD2	H2686 ND1	3.86
**Hydrogen bonding**	E99 O	Y2692 HH	1.78
	V103 H	M2668 O	2.57
	V103 O	V2670 H	2.38
	T105 H	V2670 O	2.41
	T105 O	V2672 H	2.48
	N107 HD21	G2674 O	1.64
	R113 HH21	V2684 O	1.87

Supplementary Table 3 | Residue pairs forming hydrogen bonds and salt bridges across the interface in the $FLNC_{d24}$:$HSPB7_{ACD}^{C131S}$ crystal structure.

	ID	Treatment	Sex	Age (days)	Genetic background
**Western blotting**	WKBW1.1a	Sham	M	81	C57BL/6JOlaHsd
	WARW27.1b	TAC	M	75	C57BL/6JOlaHsd
	WARW33.2a	Sham	M	85	C57BL/6JOlaHsd
	WKBW1.1d	TAC	M	81	C57BL/6JOlaHsd
	TGEK70.1e	saline	M	120	C57BL/6JOlaHsd
	TGEK63.1e	IsoPE	M	123	C57BL/6JOlaHsd
	TGEK70.2b	saline	M	119	C57BL/6JOlaHsd
	TGEK76.2b	IsoPE	M	123	C57BL/6JOlaHsd
	WARW10.1k	WT	F	74	C57BL/6J
	709	MLP KO	F	74	C57BL/6J
	WBZW1.1d	WT	F	74	C57BL/6J
	710	MLP KO	F	74	C57BL/6J
**Immunoprecipitation**	WARW33.2b	WT	M	85	C57BL/6JOlaHsd
	no ID	MLP KO	M	136	C57BL/6J

Supplementary Table 4 | Details of the mice used in the experiments show in Figure 1.

Supplementary References

1. Bolte, S. & Cordelières, F. P. A guided tour into subcellular colocalization analysis in light microscopy. *J Microsc* **224**, 213–232 (2006).

2. Dunn, K. W., Kamocka, M. M. & McDonald, J. H. A practical guide to evaluating colocalization in biological microscopy. *Am J Physiol Cell Physiol* **300**, (2011).

3. Hoffman, N. J. *et al.* Global Phosphoproteomic Analysis of Human Skeletal Muscle Reveals a Network of Exercise-Regulated Kinases and AMPK Substrates. *Cell Metab* **22**, 922–935 (2015).

4. Bouhaddou, M. *et al.* The Global Phosphorylation Landscape of SARS-CoV-2 Infection. *Cell* **182**, 685-712.e19 (2020).

5. Benesch, J. L. P. & Ruotolo, B. T. Mass spectrometry: Come of age for structural and dynamical biology. *Curr Opin Struct Biol* **21**, 641–649 (2011).
