# Supplementary material for: Filamin C dimerisation is regulated by HSPB7
Source: Nat Commun. 2025 May 1;16:4090. doi: 10.1038/s41467-025-58889-x (PMC12046049; doi:10.1038/s41467-025-58889-x)
Supplement: Supplementary file 2 — Description of Additional Supplementary Files [file 41467_2025_58889_MOESM2_ESM.pdf]

Description of Additional Supplementary Files

File Name: Supplementary Data 1

Description: Spreadsheet containing all the hydrogen-bond contact data from the MD simulations, relevant to Fig. 5 and Supplementary Fig. 7, Supplementary Fig. 8, Supplementary Fig. 9.
