# Supplementary material for: Filamin C dimerisation is regulated by HSPB7
Source: Nat Commun. 2025 May 1;16:4090. doi: 10.1038/s41467-025-58889-x (PMC12046049; doi:10.1038/s41467-025-58889-x)
Supplement: Supplementary file 4 — Reporting Summary [file 41467_2025_58889_MOESM4_ESM.pdf]

nature portfolio

Corresponding author(s): Katja Gehmlich and Justin Benesch

Last updated by author(s): 03/03/2025

Reporting Summary

Nature Portfolio wishes to improve the reproducibility of the work that we publish. This form provides structure for consistency and transparency in reporting. For further information on Nature Portfolio policies, see our Editorial Policies and the Editorial Policy Checklist.

Statistics

For all statistical analyses, confirm that the following items are present in the figure legend, table legend, main text, or Methods section.

n/a	Confirmed	
☐	☒	The exact sample size (*n*) for each experimental group/condition, given as a discrete number and unit of measurement
☐	☒	A statement on whether measurements were taken from distinct samples or whether the same sample was measured repeatedly
☐	☒	The statistical test(s) used AND whether they are one- or two-sided<br>*Only common tests should be described solely by name; describe more complex techniques in the Methods section.*
☒	☐	A description of all covariates tested
☒	☐	A description of any assumptions or corrections, such as tests of normality and adjustment for multiple comparisons
☒	☐	A full description of the statistical parameters including central tendency (e.g. means) or other basic estimates (e.g. regression coefficient) AND variation (e.g. standard deviation) or associated estimates of uncertainty (e.g. confidence intervals)
☒	☐	For null hypothesis testing, the test statistic (e.g. *F*, *t*, *r*) with confidence intervals, effect sizes, degrees of freedom and *P* value noted<br>*Give P values as exact values whenever suitable.*
☒	☐	For Bayesian analysis, information on the choice of priors and Markov chain Monte Carlo settings
☒	☐	For hierarchical and complex designs, identification of the appropriate level for tests and full reporting of outcomes
☒	☐	Estimates of effect sizes (e.g. Cohen's *d*, Pearson's *r*), indicating how they were calculated

Our web collection on statistics for biologists contains articles on many of the points above.

Software and code

Policy information about availability of computer code

Data collection	Software used to collect data is commercial; associated with the instrumentation used. All software is detailed in the methods section, and the manufacturer stated.
Data analysis	All data analysis software /code is available, either commercially (provided with the instrumentation) or freely available for academic use. The software is all detailed in the methods section, referenced appropriately.

For manuscripts utilizing custom algorithms or software that are central to the research but not yet described in published literature, software must be made available to editors and reviewers. We strongly encourage code deposition in a community repository (e.g. GitHub). See the Nature Portfolio guidelines for submitting code & software for further information.

Data

Policy information about availability of data

All manuscripts must include a data availability statement. This statement should provide the following information, where applicable:

- Accession codes, unique identifiers, or web links for publicly available datasets
- A description of any restrictions on data availability
- For clinical datasets or third party data, please ensure that the statement adheres to our policy

Data is freely available (but embargoed until acceptance of the manuscript) on the Oxford Research Archives data repository, https://ora.ox.ac.uk. The data has a unique DOI to enable easy retrieval that will be included in the manuscript.

Research involving human participants, their data, or biological material

Policy information about studies with human participants or human data. See also policy information about sex, gender (identity/presentation), and sexual orientation and race, ethnicity and racism.

Reporting on sex and gender	n/a
Reporting on race, ethnicity, or other socially relevant groupings	n/a
Population characteristics	n/a
Recruitment	n/a
Ethics oversight	n/a

Note that full information on the approval of the study protocol must also be provided in the manuscript.

Field-specific reporting

Please select the one below that is the best fit for your research. If you are not sure, read the appropriate sections before making your selection.

[x] Life sciences [ ] Behavioural & social sciences [ ] Ecological, evolutionary & environmental sciences

For a reference copy of the document with all sections, see nature.com/documents/nr-reporting-summary-flat.pdf

Life sciences study design

All studies must disclose on these points even when the disclosure is negative.

Sample size	n = 2 for in vivo mouse models; n = 1 for immunoprecipitation
Data exclusions	No data was excluded.
Replication	Mass spectrometry measurements were performed in multiplicate, as described in the methods; all the data was used.
Randomization	in vivo mouse models: random block design for treatments. Re-use of samples from previous published studies. Two samples each randomly selected for this qualitative study.
Blinding	Operators were blinded during processing and analysis of mouse samples.

Reporting for specific materials, systems and methods

We require information from authors about some types of materials, experimental systems and methods used in many studies. Here, indicate whether each material, system or method listed is relevant to your study. If you are not sure if a list item applies to your research, read the appropriate section before selecting a response.

Materials & experimental systems

n/a	Involved in the study	
[ ]	[x]	Antibodies
[x]	[ ]	Eukaryotic cell lines
[x]	[ ]	Palaeontology and archaeology
[ ]	[x]	Animals and other organisms
[x]	[ ]	Clinical data
[x]	[ ]	Dual use research of concern
[x]	[ ]	Plants

Methods

n/a	Involved in the study	
[x]	[ ]	ChIP-seq
[x]	[ ]	Flow cytometry
[x]	[ ]	MRI-based neuroimaging

Antibodies

Antibodies used	HSPB7 (15700-1-AP, Proteintech),<br>HSPB7 (NBP1-84334, Novus)<br>filamin C: FLNC antibody RR90 gift by D.O Fuerst (IF), R1899; MRC PPU (Medical Research Council Protein Phosphorylation and Ubiquitinylation Unit) Reagents and Services, University of Dundee] (WB)<br>anti-GAPDH (glyceraldehyde phosphate dehydrogenase) (ABS16, Merck)

Validation	HSBP7 - knock out cells (in-house), validation by companies and 3 publications (Proteintech) /1 publication)Novus)<br>FLNC: RR90 D. O. Fürst lab (PMID: 10658210).<br>R1899 by MRC PPU and confirmation of specificity in-house with mass spec identification after immuno-precipitation experiments<br>GAPDH: company and 10 publications on their website. We cross-validated with rabbit GAPDH antibody (Proteintech)

Animals and other research organisms

Policy information about studies involving animals; ARRIVE guidelines recommended for reporting animal research, and Sex and Gender in Research

Laboratory animals	species: mouse (Mus m.) on C57BL/6J genetic background; age, sex and sub-strain (C57BL/6J or C57BL/6JOlaHsd) of each animal stated in Table in Suppl. Material.<br>Mice (Mus musculus, on C57BL/6J background) were housed in specific pathogen–free conditions, with the only reported positives on health screening over the entire time course of these studies being for Tritrichomonas sp. and Entamoeba spp. All animals were housed at 19-21°C, at 55+/-10% humidity, in social groups (unless they had surgical interventions, then they were singly housed after the intervention). They were provided with food (Irradiated Global 16% rodent diet T26.16MI, Envigo RMS, UK, Ltd) and water ad libitum, and maintained on a 12-hour light/12-hour dark cycle (150 to 200 lux cool white light-emitting diode light, measured at the cage floor).
Wild animals	no wild animals used
Reporting on sex	This is a qualitative study. Hence only male mice were used for intervention studies (TAC and IsoPE) as they are known to have better response to interventions, less variability (caused by estrus cycle in females) and females were used for breeding.<br>For MLP KO Western blotting, females were used, as the phenotype is the same in male and female mice. Matching wildtype females were used.
Field-collected samples	no field collected samples
Ethics oversight	Experimental procedures were performed in accordance with the UK Home Office guidelines (project licenses 30/2444 and 30/2977) and approved by the institutional review board (ACER, University of Oxford, UK).

Note that full information on the approval of the study protocol must also be provided in the manuscript.

Plants

Seed stocks	n/a
Novel plant genotypes	n/a
Authentication	n/a
